# Report on Exemption in Erie County

**Published:** 1863-01

**Authors:** J. S. Trowbridge, James E. King

**Affiliations:** Examining Surgeon for Erie County; Buffalo; Examining Surgeon for Erie County; Buffalo


### REPORT ON EXEMPTION IN ERIE COUNTY.

BUFFALO, December 8th, 1862.

To Surgeon-General S. Oakley Vanderpoel, State of New York:

The undersigned, appointed Examining Surgeons of Erie County, having, by orders of the Adjutant General of the State, concluded their labors, report, that they have examined applicants for exemption from the military draft, and that they have classified complaints, rejections and exemptions, as follows:

Total number of persons applying for certificates of exemption in Erie County at large, is .................................... 4031

Total number of persons applying for certificates in County at large, and granted, is .................................... 2848

Total number of persons applying for certificates for County at large, and rejected, is .................................... 983

Total number applying for certificates in the City of Buffalo at large, is .................................... 1754

Total number of persons applying for certificates in the City of Buffalo, and rejected, is .................................... 856

Total number of persons applying for certificates in the City of Buffalo, and granted, is .................................... 1398

Certificates of exemption were given for the following diseases and disabilities:

| | |
|---|---|
| Asthma, | 44 |
| Apoplexy, | 1 |
| Angina Pectoris, | 1 |
| Abscess, | 3 |
| Aneurism, | 2 |
| Chronic Diarrhœa, | 8 |
| Castration, | 3 |
| Convulsions, | 1 |
| Cancer, | 2 |
| Chronic Cysitis, | 12 |
| " Dysentery, | 1 |
| Disease of Liver, | 13 |
| " Throat and Lungs, | 179 |
| " Kidneys, | 21 |
| " Heart, | 150 |
| " Eyes, | 41 |
| " Skin, | 11 |
| " Bowels, | 14 |
| Habitual Drunkeness, | 1 |
| Deafness, | 102 |
| Defective vision and loss of sight, | 195 |
| Defective speech, | 10 |
| Deformity of bones of Sternum, | 50 |
| Dropsy, | 7 |
| Epilepsy, | 38 |
| Fistula in Ano, | 13 |
| Fractured bones of Cranium, | 15 |
| Fevers, | 14 |
| Fistula Perineum, | 1 |
| Gottre, | 1 |
| General Debility, | 36 |
| Hemorrhoids, | 186 |
| Hernia, right inguinal, | 145 |
| Hernia, left " | 136 |
| " Umbillicalis, | 13 |
| " Abdominal, | 3 |
| " right Crural, | 20 |
| " left " | 11 |
| Hip Disease, | 16 |
| Hydrocele, | 9 |
| Injury to Limb, | 141 |
| " Joint, | 457 |
| Idiocy, | 2 |
| Loss of Teeth, | 4 |
| Lethargy, | 1 |
| Masturbation and seminal emissions, | 7 |
| Myopia, | 160 |
| Orchitis, | 8 |
| Obeisity, | 3 |
| Paralysis, | 19 |
| Polypus of Nose, | 1 |
| Spinal curvature and disease of spine, | 27 |
| Spleenitis, | 1 |
| Sciatica, | 2 |
| Scrofula, | 49 |
| Syphilis, constitutional, | 29 |
| Rheumatism, | 72 |
| Retention of testical within abdominal ring, | 2 |
| Tumors, | 13 |
| Ulcers, indolent, | 36 |
| Varicose Veins, | 105 |
| Varicocele, left side, | 41 |
| " right side, | 4 |
| Vertigo, | 2 |

The Examining Surgeons would further report, that their labors have been severe, and that in the rush of business the first two days, those examined and rejected were not registered, and that at least two hundred persons examined and rejected as having no claims for exemption, should be added to the total of applicants, making four thousand and thirty-one in all.

In the classification of diseases, as above, we would remark that most of the diseases of the heart were from the effects of acute or inflammatory rheumatism.

Especially in regard to hernia of all kinds and hemorrhoids, we would state that these diseases occurred among all classes of people, from the hard-working laborer and rugged and lusty man, to those engaged in sedentary pursuits: and that the complaints for exemptions on these grounds amounted to one-fifth of all who applied. Many, apparently sound, healthy men, applying for release from military draft, who upon a rigid examination, were discarded as unfit, excited the remarks of friends and acquaintances at their exemption, all of whom, according to the instructions of the Surgeon-General, were incapacitated. At the same time, some persons may have been exempted in the confusion of the crowds who came at the first opening of the Board to declare themselves unfit, who were not really entitled to exemption.

Under the head of injury to limbs and joints, there are 608 applicants—25 per cent. of the whole number. To account for so large a proportion, we can only base it on the ground of the City being so completely commercial and manufacturing in its character, and, therefore, the liability to accidents, by fractures and injuries must be largely in excess of those occurring in the country and agricultural communities.

Under the head of general ill-health and debility, are comprehended those whose constitutions have been broken down by recent or remote disease, those convalescing from fevers, and those suffering from dyspepsia.

Under the head of loss of limbs and joints are put down, and include loss of any limbs or joints, on hands or feet.

Of rheumatism, under which head should properly be denoted nearly all diseases of the heart, we find about eight per cent. of all applicants for exemption. We can only account for this large proportion from the fact that the surface of this county is very low, and that dampness from the lake, with its fogs, are very productive and inducive of these diseases, and are, undoubtedly, their primal cause.

The ratio of those exempted to the whole number of applicants, is as 1 to 3¼.

Ratio of those rejected to those exempted as one to two and two-fifths.

The examination of the applicants was conducted as carefully and rigidly as time, place and circumstance would allow, with a desire to perform scientifically the commission to which we were appointed, and to give even and exact justice to all.

In reviewing our labors, we think there are very few who were rejected by this Board, but what were fit for military duty, and who would, in case of their being drafted, pass any examining surgeon of the army, as sound and fit for camp.

J. S. Trowbridge, M. D.
James E. King, M. D.
*Examining Surgeons for Erie County.*

